# Propofol EC_50_ for inducing loss of consciousness in patients under combined epidural-general anesthesia or general anesthesia alone: a randomized double-blind study

**DOI:** 10.3389/fmed.2023.1194077

**Published:** 2023-11-06

**Authors:** Jiangling Wang, Yajian Shen, Wenjing Guo, Wen Zhang, Xiaoying Cui, Shunv Cai, Xinzhong Chen

**Affiliations:** ^1^Department of Anesthesia, Women’s Hospital, Zhejiang University School of Medicine, Hangzhou, China; ^2^Department of Anesthesiology, Zhejiang Cancer Hospital, Hangzhou, China

**Keywords:** epidural analgesia, anesthesia, anesthetic, propofol, EC_50_, loss of consciousness (LOC)

## Abstract

**Background:**

Combined epidural-general anesthesia (GA + EA) has been recommended as a preferred technique for both thoracic and abdominal surgery. The epidural anesthesia on the general anesthetic (GA) requirements has not been well investigated. Therefore, we conducted the present study to explore the predicted effect-site concentration of propofol (Ce_prop_) required for achieving the loss of consciousness (LOC) in 50% of patients (EC_50_) with or without epidural anesthesia.

**Methods:**

Sixty patients scheduled for gastrectomy were randomized into the GA + EA group or GA alone group to receive general anesthesia alone. Ropivacaine 0.375% was used for epidural anesthesia to achieve a sensory level of T4 or above prior to the induction of general anesthesia. The EC_50_ of predicted Ce_prop_ for LOC was determined by the up–down sequential method. The consumption of anesthetics, emergence time from anesthesia, and postoperative outcomes were also recorded and compared.

**Results:**

The EC_50_ of predicted Ce_prop_ for LOC was lower in the GA + EA group than in the GA alone group [2.97 (95% CI: 2.63–3.31) vs. 3.36 (95% CI: 3.19–3.53) μg mL^−1^, (*p* = 0.036)]. The consumption of anesthetics was lower in the GA + EA group than in the GA alone group (propofol: 0.11 ± 0.02 vs. 0.13 ± 0.02 mg kg^−1^ min^−1^, *p* = 0.014; remifentanil: 0.08 ± 0.03 vs. 0.14 ± 0.04 μg kg^−1^ min^−1^, *p* < 0.001). The emergence time was shorter in the GA + EA group than in the GA alone group (16.0 vs. 20.5 min, *p* = 0.013).

**Conclusion:**

Concomitant epidural anesthesia reduced by 15% the EC_50_ of predicted Ce_prop_ for LOC, decreased the consumptions of propofol and remifentanil during maintenance of anesthesia, and fastened recovery from anesthesia.

**Clinical trial registration:**

ClinicalTrials.gov, identifier: NCT05124704.

## Introduction

Combined epidural-general anesthesia (GA + EA) has been well accepted to be the preferred anesthetic technique for major upper abdominal and thoracic surgeries (1–3) due to better pain control, rapid recovery, improved postoperative outcomes, lower morbidity, and mortality compared to general anesthesia (GA) alone (4–7). In addition, neuraxial (epidural) anesthesia has been shown to have direct sedative effects and to decrease the requirements of volatile anesthetics or opioids for adequate depth of anesthesia and analgesia during general anesthesia (8, 9).

We hypothesized that the effect-site concentration of propofol required for loss of consciousness (LOC) could be decreased by concomitant epidural anesthesia during the induction of general anesthesia.

Therefore, we conducted this prospective, randomized, and double-blind trial to investigate the median (50%) effective effect-site concentration (EC_50_) of propofol for inducing LOC in patients undergoing open gastrectomy under combined epidural-general anesthesia and general anesthesia alone using the up–down sequential allocation method. We also investigated and compared the consumption of anesthetics, hemodynamic status, emergence time, and postoperative outcomes between combined epidural-general anesthesia and general anesthesia alone.

## Methods

### Study population and randomization

This study was approved by the Ethics Committee of Zhejiang Cancer Hospital prior to patient enrolment (IRB-2021-214, date of approval: 1 July 2021) and registered at ClinicalTrials.gov (identifier: NCT05124704, registration date: 11 October 2021, principal investigator: JW). We followed the consolidated Standards of Reporting Trials (CONSORT) statement when conducting and reporting this trial. This study was conducted between 20 November 2021 and 25 May 2022 at the Department of Anesthesiology, Zhejiang Cancer Hospital.

After obtaining written informed consent, 60 ASA physical states II or III patients with gastric cancer aged 18–75 years scheduled for open gastrectomy were enrolled in this study. The exclusion criteria were as follows: contraindications to epidural puncture or catheter placement; chronic or acute (within 48 h) intake of psychotropic drugs, benzodiazepines, anticonvulsants, or opioids; alcoholism; hepatic, renal, neurological, or other organ dysfunction; allergy to any drugs used in this study; or refusal to receive epidural puncture.

The eligible patients were randomized to one of the two groups with different anesthesia protocols: the GA + EA group (combined epidural-general anesthesia) or the GA alone group (general anesthesia alone). Randomization was performed by a research assistant who did not take part in the study using computer-generated numbers (Microsoft Excel for MAC, Microsoft, Redmond, WA, United States). The randomization codes were concealed in numbered, sealed opaque envelopes, one of which was opened for each patient.

### Before induction of anesthesia

No premedication was given. Upon arrival in the operating theater, standard monitoring including electrocardiogram (ECG), pulse oximetry (SpO_2_), invasive artery blood pressure (IBP) via the left radial artery, bispectral index (BIS), and end-tidal carbon dioxide (EtCO_2_) was applied. A central venous catheter was placed in the right internal jugular vein and commenced an infusion of 37°C Ringer’s lactate solution at a rate of 10 mL kg^−1^ h^−1^.

### Induction of anesthesia

Prior to general anesthesia induction, all patients had an epidural catheter placed in the left lateral position. Epidural anesthesia was performed based on the usual procedure in our center and procedures described before (10). After skin infiltration with lidocaine, epidural puncture was performed with a 16-gauge Tuohy needle at the estimated T8–T9 vertebral interspace using a loss-of-resistance to saline technique and then inserted a nylon multiport catheter 4 cm into the epidural space with the needle orifice oriented cephalad.

An anesthesia nurse who was not involved in the study prepared the epidural medication (normal saline or 0.375% ropivacaine) in a 20 mL syringe. A test dose of 3 mL of the epidural medication (normal saline for the GA group and 0.375% ropivacaine for the GA + EA group) was given to exclude an intrathecal placement of the epidural catheter. After 4 min, in the absence of significant sensory or motor blockade, the patient received an extra 5 to 8 mL (depending on the height and weight) of the epidural medication (normal saline for the GA alone group and 0.375% ropivacaine for the GA + EA group) through the epidural catheter. The level of sensory blockade was assessed bilaterally in the anterior axillary line by pinprick 15 min after epidural injection. The upper and lower limits of the block level were recorded. If the upper block level reached T4 or above, but did not exceed T3, the epidural infusion was then maintained with 0.375% ropivacaine at an infusion rate of 4–6 mL h^−1^.

General anesthesia was induced with propofol oxycodone and rocuronium by the fixed attending anesthesiologists (JW, YS, and WG) who were blinded to the patient grouping and epidural administration. Propofol was administered via a target-controlled infusion (TCI) (Base Premea, Ochestraw, Fresenius Company, Bre’zins France) with the pharmacokinetic model of Schnider et al. (11).

To explore the EC_50_ of propofol-inducing LOC (defined as the loss of response to verbal commands) in patients with or without prior epidural anesthesia, the administration of propofol for each patient was applied according to that applied in our previous study (12) using the up–down sequential allocation method (13). The initial TCI Ce_prop_ for the first patient of each group was set at 3.5 μg mL^−1^. The initial TCI Ce_prop_ for the next patient was determined by the response of the previous patient to the initial dose of propofol administered. A positive response was defined as LOC occurring within 4 min of TCI propofol, and a negative response was defined as no LOC within 4 min of TCI propofol. If a positive response happened, the initial TCI Ce_prop_ was decreased by 0.5 ug mL^−1^ for the next patient in the same group. If a negative response happened, the initial TCI Ce_prop_ was increased by 0.5 μg mL^−1^ for the next patients. The TCI Ce_prop_ for the negative patient would increase by 0.5 μg mL^−1^ stepwise at 4 min intervals until the patient showed LOC. After LOC, an intravenous bolus of 0.25 mg kg^−1^ oxycodone was administered, and then 0.6 mg kg^−1^ rocuronium was given to facilitate tracheal intubation (14).

### Maintenance of anesthesia

Anesthesia was maintained with propofol and remifentanil. Ce_prop_ was adjusted immediately after intubation by increasing or decreasing in steps of 0.5 μg mL^−1^ to keep the BIS value between 40 and 60 throughout the surgery. The lowest Ce_prop_ was 2.0 μg mL^−1^ in order to prevent intraoperative awareness (12). In the meantime, intravenous infusion of remifentanil began at a rate of 0.1 μg kg^−1^ min^−1^ and was then adjusted by increasing or decreasing in steps by 0.05 μg kg^−1^ min^−1^ to keep adequate anesthesia. The criteria of inadequate anesthesia were as follows (15): (1) hypertension: a mean blood pressure (MAP) >120% of baseline or >100 mmHg; (2) tachycardia: heart rate >90 beats·min^−1^; (3) somatic arousal: signs of coughing, chewing, and grimacing; and (4) somatic response: purposeful movement. The maximum infusion rate was 0.25 μg kg^−1^ min^−1^. Hypotension, defined as a MAP <80% of baseline or <60 mmHg, was treated initially by speeding intravenous infusion and decreasing the remifentanil infusion rate by 0.05 μg kg^−1^ min^−1^ in a stepwise manner until the minimum rate of 0.05 μg kg^−1^ min^−1^, and finally, ephedrine, phenylephrine, or metaraminol were given. Bradycardia was treated with intravenous atropine 0.5 mg.

### Recovery period

Both propofol and remifentanil infusions were discontinued when the final surgical suture was completed. Emergence time from anesthesia was assessed by measuring the duration between the time of discontinuation of anesthetics and the time of spontaneous opening of eyes. Patient-controlled epidural analgesia (PCEA) with 0.175% ropivacaine mixed with 0.7 μg mL^−1^ sufentanil was applied for postoperative pain for all patients. In the postanesthesia care unit (PACU), the modified Aldrete-Score, postoperative nausea and vomiting, pain intensity (visual analog scale of 0–10, 0 = no pain, 10 = worst pain imaginable), and other side effects were recorded. All patients were visited on the 1st day postoperatively to check for adverse effects of anesthesia, and patient satisfaction with the anesthesia procedure was assessed using a scale of 0–10 rating (0 = completely dissatisfied and 10 = completely satisfied) and the quality of postoperative analgesia with PCEA using the variables such as pain VAS and the number of total PCE presses and effective presses.

### Statistical analysis

The sample size of 30 patients per group was determined based on the results of the previous simulation study that suggested that 20 to 40 subjects or six crossovers were sufficient to provide a stable estimate of the EC_50_ calculated by using the modified Dixon up–down method for most realistic scenarios (16).

Continuous variables were tested for normality using the D’Agostino and Pearson tests. Variables with normal distribution were presented as the mean ± standard deviation (SD), and intergroup comparisons were performed using student’s *t*-test. Variables with non-normal distribution were presented as the median and interquartile range (IQR), and intergroup comparisons were performed using the Mann–Whitney *U*-test. Categorical variables were presented as number (%) and were analyzed using the chi-square test.

The EC_50_ and EC_95_ values for EC_50_ of propofol were determined by calculating the mean of the midpoints of pairs of Ce_prop_ administered in successive patients in which a positive response was followed by a negative response or a negative response was followed by a positive response (turning points) according to the modified up-and-down allocation method as described previously. At least six pairs of negative–positive responses were needed in each group for the final analysis (13, 17). The 95% confidence interval (CI) and SD for the EC_50_ values were calculated using the method suggested by Choi (17). Probit regression analysis was applied as a backup and sensitivity test by analyzing tallied numbers of positive patient and negative patients for each dose category for each group; estimates of the EC_50_ for propofol in each group were obtained and the difference between the two groups was quantified by calculating the relative mean potency with 95% CI. Emergence time was analyzed by using the Kaplan–Meier log-rank survival analysis to compare the cumulative probability of patients remaining unconscious after the discontinuation of propofol.

GraphPad Prism software version 5.0 (GraphPad Software Inc., San Diego, CA, United States), SPSS version 22.0 (SPSS, Inc., Chicago, IL, United States), and R package version 0.1.1 were used for statistical analysis. A *p*-value of <0.05 was considered to be statistically significant.

## Results

### Patient characteristics

**Figure 1:**
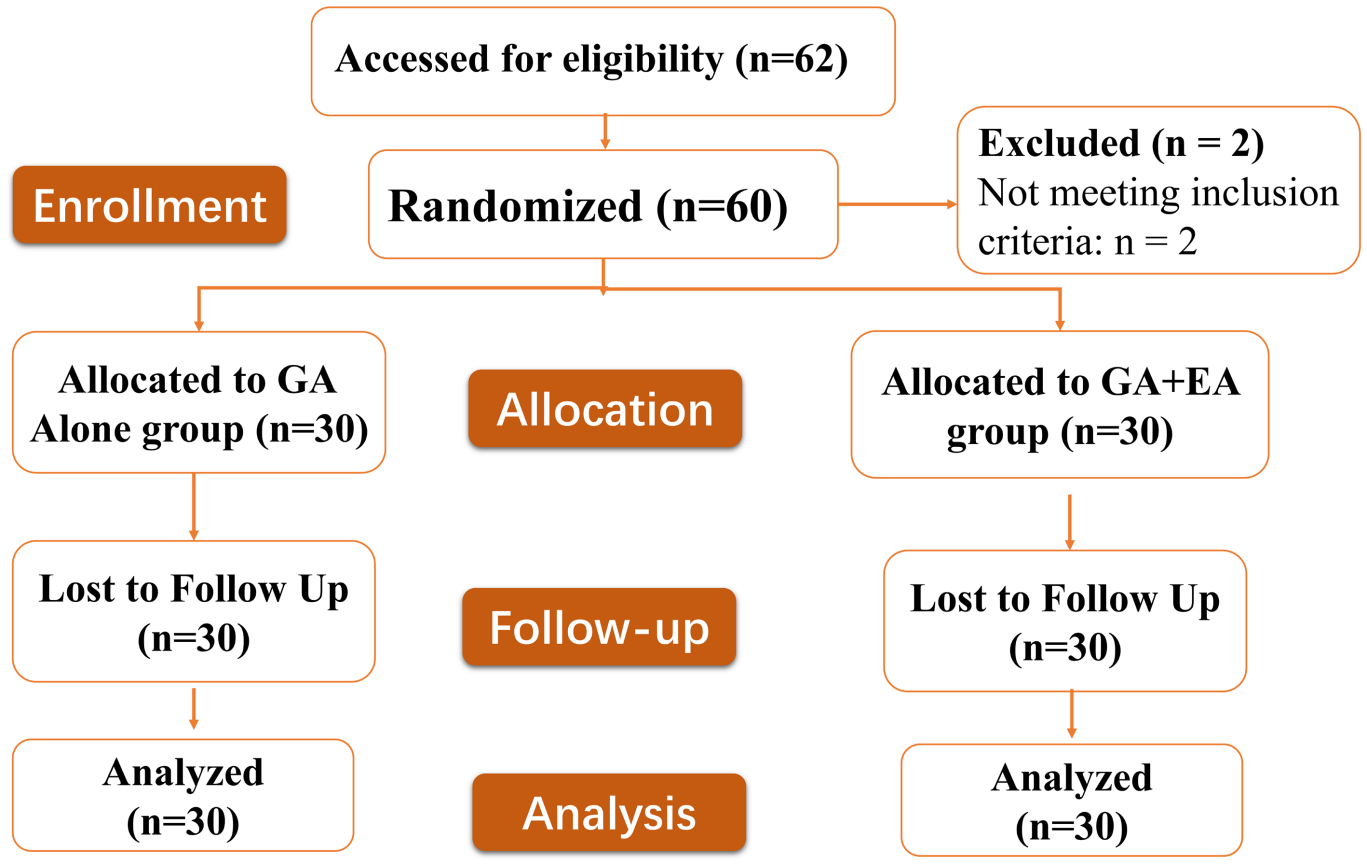
Consolidated Standards of Reporting Trials (CONSORT) flow diagram defining patient assessment and enrolment numbers in the study.

The consolidated standard of reporting trials (CONSORT) flow diagram is shown in Figure 1. Sixty-two patients were screened for eligibility, and two patients did not meet the inclusion criteria. A total of 60 patients were randomized into one of two groups (*n* = 30 each) and included in the final analysis. Baseline demographic and surgical characteristics were comparable between groups (Table 1).

**Table 1:** Patient characteristics.

	GA alone group (*n* = 30)	GA + EA group (*n* = 30)	Mean difference or RR (estimate 95% CI)	*p*-value
Age (year)	59.6 ± 11.0	59.8 ± 11.7		0.96
Male (%)	16 (53.3)	21 (70)	0.49 (0.17–1.41)	0.29
Weight (kg)	57.3 ± 13.6	61.3 ± 8.9		0.18
Height (cm)	163.3 ± 1.4	164.3 ± 1.1		0.58
ASA physical status, II/III	22/8	16/14	2.1 (0.8–7.1)	0.18
Albumin (g/L)	40.49 ± 4.73	40.25 ± 3.56		0.82
Surgery duration (min)	150.0 (130.0, 163.3)	138.0 (102.8, 169.3)		0.28
Anesthesia duration (min)	160.0 (135.8, 176.0)	143.5 (115.0, 180.0)		0.28
Propofol (mg kg^−1^ min^−1^)	0.13 (0.02)	0.11 (0.02)		0.014
Remifentanil (ug kg^−1^ min^−1^)	0.14 (0.04)	0.08 (0.03)		<0.0001

Data are presented as mean ± SD or median (IQR). ASA, American Society of Anesthesiologists; SD, standard deviation; IQR, interquartile range.

### EC_50_ and EC_95_ of propofol for LOC

**Figure 2:**
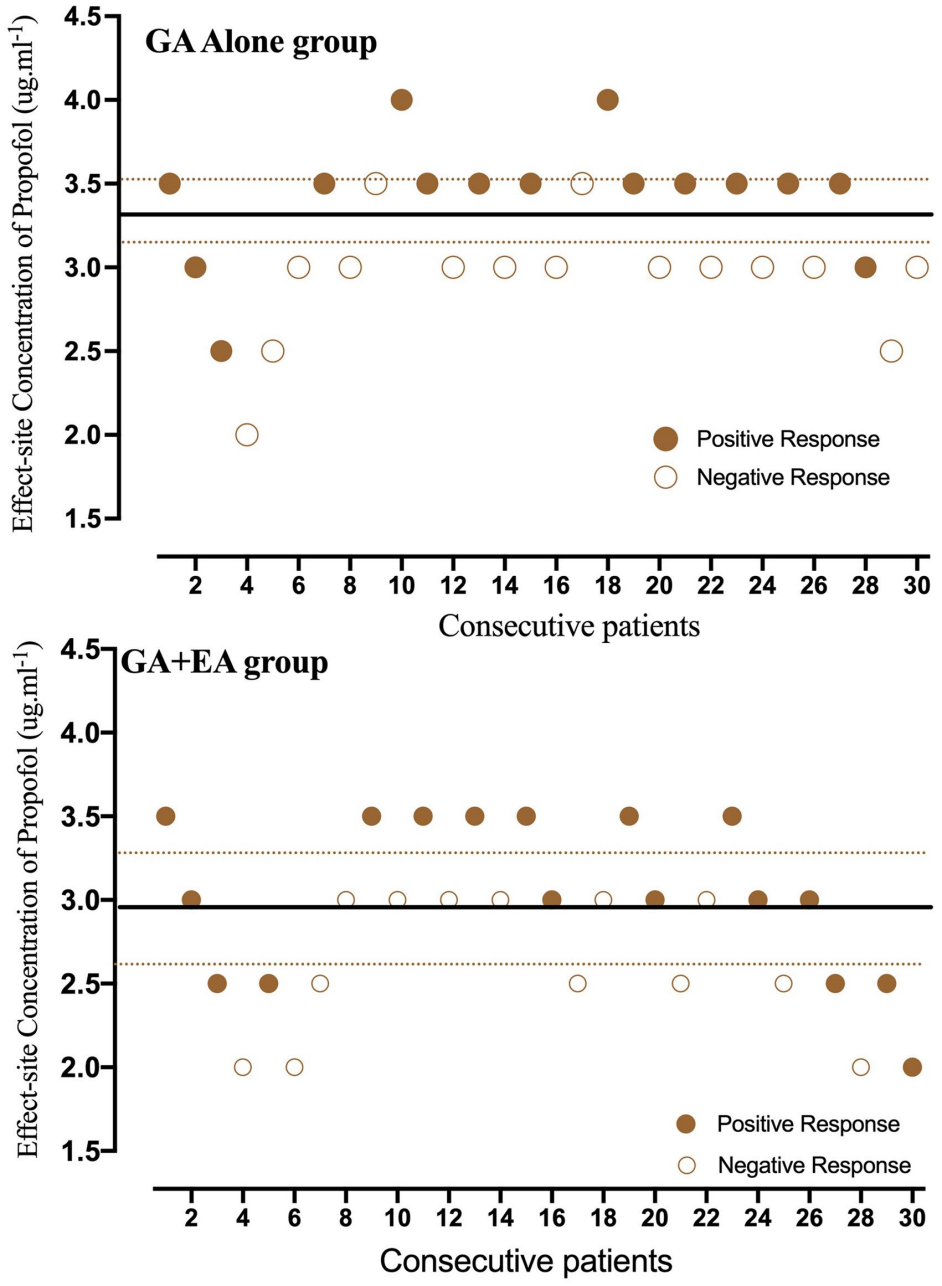
Individual responses to propofol at corresponding effect-site concentrations (Ce_prop_). Unfilled circles represent a negative response to the corresponding Ce_prop_ for achieving loss of consciousness (LOC). Fill circles represent a positive response to the corresponding Ce_prop_ for achieving LOC. Solid lines represent the mean effective concentration (EC_50_) of propofol, and dashed lines represent the 95% confidence interval.

Individual responses to propofol at corresponding Ce_prop_ are shown in Figure 2. Nine pairs of negative–positive responses in each group were included in this study. The EC_50_ of propofol for LOC, determined using Dixon’s up–down method, was lower in the GA + EA group [2.97 (95% CI: 2.63–3.31) μg mL^−1^] compared with the GA alone group [3.36 (95% CI: 3.19–3.53) μg mL^−1^] (*p* = 0.036). The EC_50_ and EC_95_ values of propofol calculated using probit regression analysis were 2.71 (95% CI: 2.32–3.04) μg mL^−1^ and 3.71 (95% CI: 3.31–4.86) μg mL^−1^, respectively, in the GA + EA group, and 3.19 (95% CI: 2.83–3.54) μg mL^−1^ and 4.19 (95% CI: 3.76–5.42) μg mL^−1^, respectively, in the GA alone group. The relative mean potency for propofol-inducing LOC in the GA alone group versus the GA + EA group was 0.85. In addition, the predicted Ce_prop_ at the time of LOC was significantly lower in the GA + EA group, 1.97 (95% CI: 1.75–2.16) μg mL^−1^, compared with the GA alone group, 2.63 (95% CI: 2.49–2.76) μg mL^−1^ (*p* < 0.001).

### Consumption of propofol and remifentanil

Both propofol and remifentanil consumptions (average normalized infusion rate which refers to the total drug used/duration between induction and discontinuation of the drug/weight) were significantly lower in the GA + EA group compared with the GA alone group (propofol: 0.11 ± 0.02 mg kg^−1^ min^−1^ vs. 0.13 ± 0.02 mg kg^−1^ min^−1^, *p* = 0.014;) (remifentanil: 0.08 ± 0.03 μg kg^−1^ min^−1^ vs. 0.14 ± 0.04 μg kg^−1^ min^−1^ *p* < 0.001).

### Emergence time

**Figure 3:**
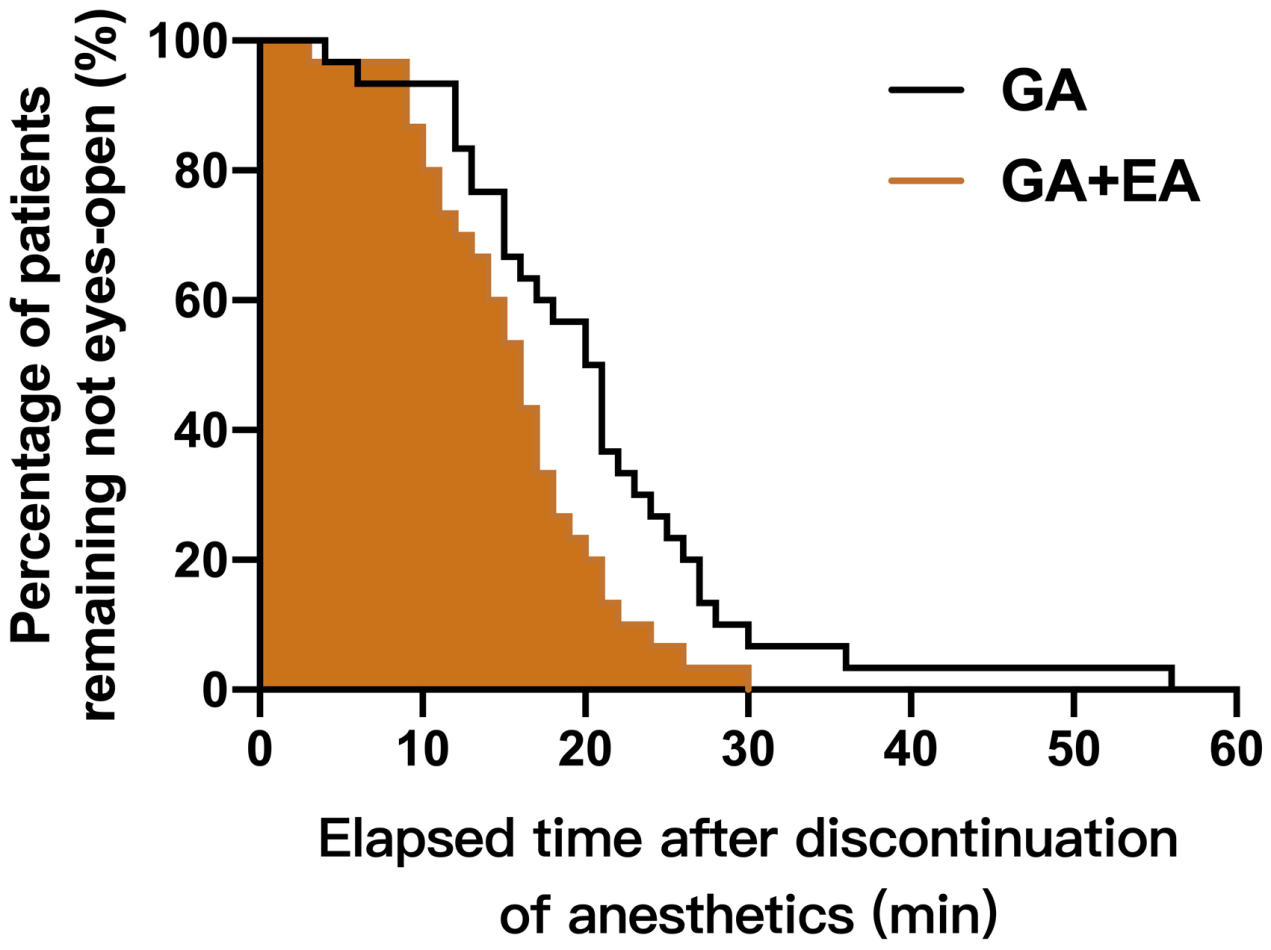
Cumulative percentages of patients remaining unconscious after discontinuation of propofol and remifentanil infusion in the GA + EA group (brown line, shaded area) and in the GA alone group (black line, empty area), using the Kaplan–Meier survival analysis.

The emergence time from anesthesia was significantly shorter in the GA + EA group [16.0 (IQR: 11.0–19.3) min] compared with the GA alone group [20.5 (IQR: 14.5–25.3) min] using the Kaplan–Meier log-rank survival analysis (*p* = 0.013). The cumulative percentages of patients remaining unconscious after discontinuation of anesthetics infusion for both groups are shown in Figure 3.

### Perioperative side effects and pain control

The incidence of hypotension during the maintenance of anesthesia was higher in the GA + EA group than in the GA alone group (*p* = 0.29). The pain score in the PACU was lower in the GA + EA group than that in the GA alone group (*p* < 0.001). The total numbers and the effective numbers of PCEA press on the 1st day postoperatively were lower in the GA + EA group than in the GA alone group (all *p* < 0.05). The incidence of postoperative side effects, such as hypotension, nausea, and vomiting, and also patient satisfaction score and time to first flatus were comparable between groups (all *p* > 0.05) (Table 2).

**Table 2:** Side effects, patient satisfaction, and postoperative pain.

	GA alone group (*n* = 30)	GA + EA group (*n* = 30)	Mean difference or RR (estimate 95% CI)	*p*-value
*Intraoperative period*				
Hypotension [*n* (%)]	16 (53.3)	21 (70.0)	2.04 (0.71–5.90)	0.29
*Postoperative period*				
Nausea [*n* (%)]	5 (16.7)	3 (10.0)	0.56 (0.12–2.57)	0.71
Vomiting [*n* (%)]	1 (3.3)	0 (0)	0.97 (0.91–1.03)	1.00
Hypotension [*n* (%)]	1 (3.3)	2 (6.7)	2.07 (0.18–24.15)	1.00
Pain score (VAS) in PACU	3.0 (1.0, 4.0)	0.0 (0.0, 1.0)		<0.001
Total number of PCEA press	6.2 (3.9, 8.5)	3.7 (2.1, 5.4)		0.04
Effective number of PCEA press	6.0 (3.8, 8.1)	3.7 (2.0, 5.3)		0.03
Patient satisfaction score	9.0 (9.0, 9.0)	9.0 (9.0, 9.0)		0.66
Modified aldrete score	9.0 (8.0, 9.0)	9.0 (9.0, 9.0)		0.59

Data are presented as *n* (%), mean ± SD or median (IQR). VAS, visual analog score; SD, standard deviation; IQR, interquartile range.

## Discussion

In this prospective, randomized, double-blind study, using the up–down sequential method, we mainly demonstrated that the predicted effect-site concentration of propofol (Ce_prop_) required for achieving loss of consciousness (LOC) in 50% of patients (EC_50_) was significantly lower in patients with prior thoracic epidural anesthesia (TEA) with 0.375% ropivacaine (GA + EA group) than in patients without prior TEA (GA alone group). The magnitude effect of TEA, the difference between the calculated EC_50_ of propofol in two groups, was 15% in the probit regression method and 12% in the up–down method. In addition, we also demonstrated that the patients in the GA + EA group had lower consumptions of both propofol and remifentanil during maintenance of anesthesia, shorter emergence time from anesthesia, and better postoperative pain control compared with the patients in the GA alone group.

The mechanisms by which the epidural local anesthetic reduced the requirements of propofol for LOC are not yet clear. The first explanation for this sparing effect might be the reduction in afferent input induced by the epidural block; in other words, neuraxial anesthesia could result in direct sedative effects (8, 18). The second explanation might be the direct effect of systemic local anesthetic absorbed from epidural space. Intravenous lidocaine has sedative or analgesia effects which was validated by our previous study (19) and other studies (20, 21). However, the plasma levels of ropivacaine absorbed from epidural space were found to be very low (22). The sedative effect of systemic ropivacaine could be limited. On the other side, Sitsen et al. (23) found that in the presence of an epidural blockade of 20 segments using ropivacaine, blood propofol concentrations are elevated by approximately 30% due to a reduced propofol elimination clearance. Cardiac output and serum albumin possibly contribute to the effect of propofol. The MAP and heart rate as well as the albumin level (latest before surgery) fluctuation were not significantly different between the two groups (Supplementary material). The measurement of plasma concentration of ropivacaine needs to be conducted in further studies to confirm the conclusions. The third explanation might be the alteration of propofol pharmacokinetics produced by epidural anesthesia. The Ce_prop_ has not been measured but calculated according to the pharmacokinetic model of propofol described by Schnider et al. (11) in the present study. The real Ce_prop_ was very likely to be lower than the predicted Ce_prop_ in the presence of epidural anesthesia. The fourth explanation might be the effect of the rostral spread of ropivacaine through the cerebrospinal fluid with direct actions on the brain.

The benefits of epidural anesthesia have persisted since its first application. Risks and side effects were rare and have gradually decreased with the development of technology and medicine. Epidural anesthesia with local anesthetics was demonstrated to reduce the sevoflurane requirements for adequate depth of anesthesia during induction or maintenance of general anesthesia with a reduction magnitude of over 30% (9, 24, 25). However, the effects of epidural anesthesia on the propofol requirements for achieving LOC have not been well investigated. Our results showed that epidural ropivacaine significantly reduced propofol EC_50_ for LOC, propofol, and remifentanil consumptions during maintenance, which may contribute to shorter emergence time. Results are consistent with previous studies (26, 27); epidural anesthesia enhances the intraoperative quality, provides further evidence of the synergic effects on sedation, facilitates early recovery after surgery, is more likely to avoid nausea and/or vomiting, and relieves the pain and promotes early activity postoperatively; these benefits may be a reference for clinical management when this procedure was applied.

Side effects, such as postoperative pain, nausea, and vomiting, can delay wound healing, prolong eating time, and affect recovery after surgery (27, 28). The use of large-dose opioids increases the incidence of nausea and vomiting postoperatively (29–31). In our study, the dosage of opioids was significantly lower, emergence time was significantly shorter, and patients experienced lower pain intensity postoperatively in the GA + EA group, which may contribute to the lower incidence of nausea and vomiting as well as better recovery after surgery in these patients. These findings further provide evidence of the first choice of combined epidural to general anesthesia in open abdominal surgery.

Our study has several limitations. First, we did not measure the plasma concentrations of propofol (Cp_prop_) and detect the EC_95_ of propofol for LOC. Considering the fact that the administration of propofol using the TCI technique is very popular, the results of predicted Ce_prop_ required for LOC found in the present study could be a reference for clinical practice. Consequently, we assessed the sensory block level 15 min after epidural infusion. This time may not be long enough for the local anesthetics to cease its maximal rostral spread after epidural infusion; thus, the actual sensory level during induction of anesthesia might be higher than we recorded. Next, epidural anesthesia is an invasive procedure that may have some risks and side effects such as low blood pressure, back pain, sympathetic nerve blockade, damage to nerves at the injection site, discitis, osteomyelitis, or meningitis. Finally, we measured the BIS value but did not record and analyze this variable. Further studies are needed to be conducted to minimize these limitations.

## Conclusion

Under the conditions of the present study, concomitant epidural anesthesia with ropivacaine achieving a sensory block level of T4 or above could reduce 15% of the EC_50_ of predicted effect-site concentration of propofol required for achieving LOC during induction of general anesthesia in patients with gastric cancer. In addition, patients in the GA + EA group had lower consumptions of propofol and remifentanil during maintenance of anesthesia, faster recovery from anesthesia, and improved postoperative pain control compared with the patients in the GA alone group.

## Data availability statement

The datasets presented in this study can be found in online repositories. The names of the repository/repositories and accession number(s) can be found at: https://clinicaltrials.gov/ct2/show/NCT05124704?term=NCT05124704&draw=2&rank=1.

## Ethics statement

The studies involving humans were approved by Ethics Committee of Zhejiang Cancer Hospital. The studies were conducted in accordance with the local legislation and institutional requirements. The participants provided their written informed consent to participate in this study.

## Author contributions

JW and XCh: study design, data analysis, and manuscript preparation. JW, YS, WG, XCu, WZ, and SC: conducting the study. WG and WZ: data collection. XCh: random code generation. All authors contributed to the article and approved the submitted version.

## References

[1] Chou R Gordon DB de Leon-Casasola OA Rosenberg JM Bickler S Brennan T. Management of postoperative pain: a clinical practice guideline from the American Pain Society, the American Society of Regional Anesthesia and Pain Medicine, and the American Society of Anesthesiologists’ Committee on Regional Anesthesia, Executive Committee, and Administrative Council. J Pain. (2016) 17:131–57. doi: 10.1016/j.jpain.2015.12.008 26827847

[2] Melloul E Lassen K Roulin D Grass F Perinel J Adham M. Guidelines for perioperative care for pancreatoduodenectomy: enhanced recovery after surgery (ERAS) recommendations 2019. World J Surg. (2020) 44:2056–84. doi: 10.1007/s00268-020-05462-w 32161987

[3] Von Dossow V Welte M Zaune U Martin E Walter M Ruckert J. Thoracic epidural anesthesia combined with general anesthesia: the preferred anesthetic technique for thoracic surgery. Anesth Analg. (2001) 92:848–54. doi: 10.1097/00000539-200104000-00010 11273913

[4] Buggy DJ Smith G. Epidural anaesthesia and analgesia: better outcome after major surgery? Growing evidence suggests so. BMJ. (1999) 319:530–1. doi: 10.1136/bmj.319.7209.530 10463878 PMC1116422

[5] Salata K Abdallah FW Hussain MA de Mestral C Greco E Aljabri B. Short-term outcomes of combined neuraxial and general anaesthesia versus general anaesthesia alone for elective open abdominal aortic aneurysm repair: retrospective population-based cohort study^†^. Br J Anaesth. (2020) 124:544–52. doi: 10.1016/j.bja.2020.01.018 32216957

[6] Popping DM Elia N Van Aken HK Marret E Schug SA Kranke P. Impact of epidural analgesia on mortality and morbidity after surgery: systematic review and meta-analysis of randomized controlled trials. Ann Surg. (2014) 259:1056–67. doi: 10.1097/SLA.0000000000000237 24096762

[7] Rigg JR Jamrozik K Myles PS Silbert BS Peyton PJ Parsons RW. Epidural anaesthesia and analgesia and outcome of major surgery: a randomised trial. Lancet. (2002) 359:1276–82. doi: 10.1016/S0140-6736(02)08266-1 11965272

[8] Gentili M Huu PC Enel D Hollande J Bonnet F. Sedation depends on the level of sensory block induced by spinal anaesthesia. Br J Anaesth. (1998) 81:970–1. doi: 10.1093/bja/81.6.970 10211030

[9] Hodgson PS Liu SS. Epidural lidocaine decreases sevoflurane requirement for adequate depth of anesthesia as measured by the bispectral index monitor. Anesthesiology. (2001) 94:799–803. doi: 10.1097/00000542-200105000-00018 11388531

[10] Panousis P Heller AR Koch T Litz RJ. Epidural ropivacaine concentrations for intraoperative analgesia during major upper abdominal surgery: a prospective, randomized, double-blinded, placebo-controlled study. Anesth Analg. (2009) 108:1971–6. doi: 10.1213/ane.0b013e3181a2a301 19448234

[11] Schnider TW Minto CF Gambus PL Andresen C Goodale DB Shafer SL. The influence of method of administration and covariates on the pharmacokinetics of propofol in adult volunteers. Anesthesiology. (1998) 88:1170–82. doi: 10.1097/00000542-199805000-00006 9605675

[12] Fu F Chen X Feng Y Shen Y Feng Z Bein B. Propofol EC_50_ for inducing loss of consciousness is lower in the luteal phase of the menstrual cycle. Br J Anaesth. (2014) 112:506–13. doi: 10.1093/bja/aet383 24285693

[13] Dixon WJ. Staircase bioassay: the up-and-down method. Neurosci Biobehav Rev. (1991) 15:47–50. doi: 10.1016/s0149-7634(05)80090-9 2052197

[14] Jones RK Caldwell JE Brull SJ Soto RG. Reversal of profound rocuronium-induced blockade with sugammadex: a randomized comparison with neostigmine. Anesthesiology. (2008) 109:816–24. doi: 10.1097/ALN.0b013e31818a3fee 18946293

[15] Chen X Thee C Gruenewald M Wnent J Illies C Hoecker J. Comparison of surgical stress index-guided analgesia with standard clinical practice during routine general anesthesia: a pilot study. Anesthesiology. (2010) 112:1175–83. doi: 10.1097/ALN.0b013e3181d3d641 20418698

[16] Paul M Fisher DM. Are estimates of MAC reliable? Anesthesiology. (2001) 95:1362–70. doi: 10.1097/00000542-200112000-00014 11748393

[17] Choi SC. Interval estimation of the LD50 based on an up-and-down experiment. Biometrics. (1990) 46:485–92. 2364133

[18] Tverskoy M Fleyshman G Bachrak L Ben-Shlomo I. Effect of bupivacaine-induced spinal block on the hypnotic requirement of propofol. Anaesthesia. (1996) 51:652–3. doi: 10.1111/j.1365-2044.1996.tb07847.x 8758157

[19] Xu L Wang C Dai S Shen J Chen X Ngan Kee WD. Intravenous lidocaine attenuates response to cervical dilation for hysteroscopy: a randomised controlled trial. Br J Anaesth. (2021) 127:e166–8. doi: 10.1016/j.bja.2021.07.020 34420685

[20] Hans GA Lauwick SM Kaba A Bonhomme V Struys MM Hans PC. Intravenous lidocaine infusion reduces bispectral index-guided requirements of propofol only during surgical stimulation. Br J Anaesth. (2010) 105:471–9. doi: 10.1093/bja/aeq189 20650919

[21] Dunn LK Durieux ME. Perioperative use of intravenous lidocaine. Anesthesiology. (2017) 126:729–37. doi: 10.1097/ALN.0000000000001527 28114177

[22] Emanuelsson BMPJ Alm C Heller A Gustafsson LL. Systemic absorption and block after epidural injection of ropivacaine in healthy volunteers. Anesthesiology. (1997) 87:1309. doi: 10.1097/00000542-199712000-00008 9416714

[23] Sitsen E Olofsen E Lesman A Dahan A Vuyk J. Epidural blockade affects the pharmacokinetics of propofol in surgical patients. Anesth Analg. (2016) 122:1341–9. doi: 10.1213/ANE.0000000000001090 26649908

[24] Reinoso-Barbero F Martinez-Garcia E Hernandez-Gancedo MC Simon AM. The effect of epidural bupivacaine on maintenance requirements of sevoflurane evaluated by bispectral index in children. Eur J Anaesthesiol. (2006) 23:460–4. doi: 10.1017/S0265021506000330 16507194

[25] Ben-David B Vaida S Gaitini L. The influence of high spinal anesthesia on sensitivity to midazolam sedation. Anesth Analg. (1995) 81:525–8. doi: 10.1097/00000539-199509000-00017 7653816

[26] Dumans-Nizard V Le Guen M Sage E Chazot T Fischler M Liu N. Thoracic epidural analgesia with levobupivacaine reduces remifentanil and propofol consumption evaluated by closed-loop titration guided by the bispectral index: a double-blind placebo-controlled study. Anesth Analg. (2017) 125:635–42. doi: 10.1213/ANE.0000000000001996 28537969

[27] Liu Q Lin JY Zhang YF Zhu N Wang GQ Wang S. Effects of epidural combined with general anesthesia versus general anesthesia on quality of recovery of elderly patients undergoing laparoscopic radical resection of colorectal cancer: a prospective randomized trial. J Clin Anesth. (2020) 62:109742. doi: 10.1016/j.jclinane.2020.109742 32088534

[28] Chen WK Ren L Wei Y Zhu DX Miao CH Xu JM. General anesthesia combined with epidural anesthesia ameliorates the effect of fast-track surgery by mitigating immunosuppression and facilitating intestinal functional recovery in colon cancer patients. Int J Color Dis. (2015) 30:475–81. doi: 10.1007/s00384-014-2098-1 25579161

[29] Skolnik A Gan TJ. Update on the management of postoperative nausea and vomiting. Curr Opin Anaesthesiol. (2014) 27:605–9. doi: 10.1097/ACO.0000000000000128 25225824

[30] Cao X White PF Ma H. An update on the management of postoperative nausea and vomiting. J Anesth. (2017) 31:617–26. doi: 10.1007/s00540-017-2363-x 28455599

[31] Grape S Usmanova I Kirkham KR Albrecht E. Intravenous dexamethasone for prophylaxis of postoperative nausea and vomiting after administration of long-acting neuraxial opioids: a systematic review and meta-analysis. Anaesthesia. (2018) 73:480–9. doi: 10.1111/anae.14166 29226971

