# Supplementary material for: Propofol EC50 for inducing loss of consciousness in patients under combined epidural-general anesthesia or general anesthesia alone: a randomized double-blind study
Source: Front Med (Lausanne). 2023 Nov 6;10:1194077. doi: 10.3389/fmed.2023.1194077 (PMC10661411; doi:10.3389/fmed.2023.1194077)
Supplement: Supplementary file 1 [file Data_Sheet_1.docx]

**Supplement figure 1**


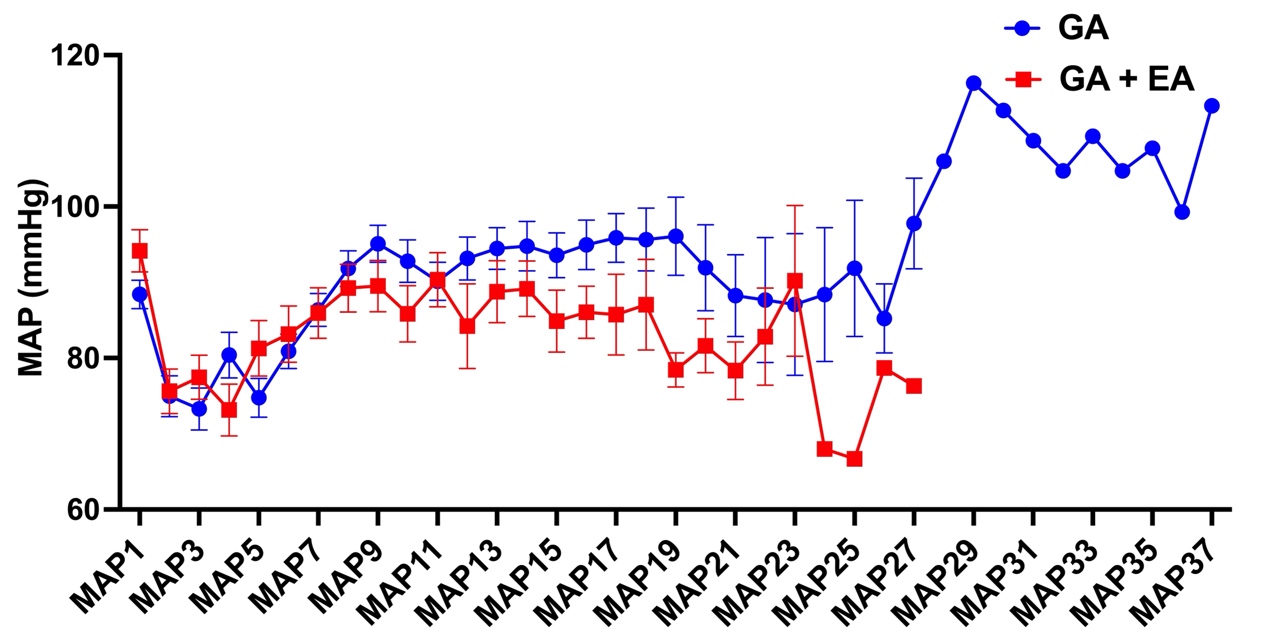


Supplement figure 1: MAP fluctuation between two groups

Data were recorded at 5min interval, MAP1 was the baseline value, MAP2 was the value right after induction. Most of the patient were finished their surgery within 200 mins. Four patients in GA group and 2 patients in the GA+EA group have exceeded 200 mins.

**Supplement figure 2**


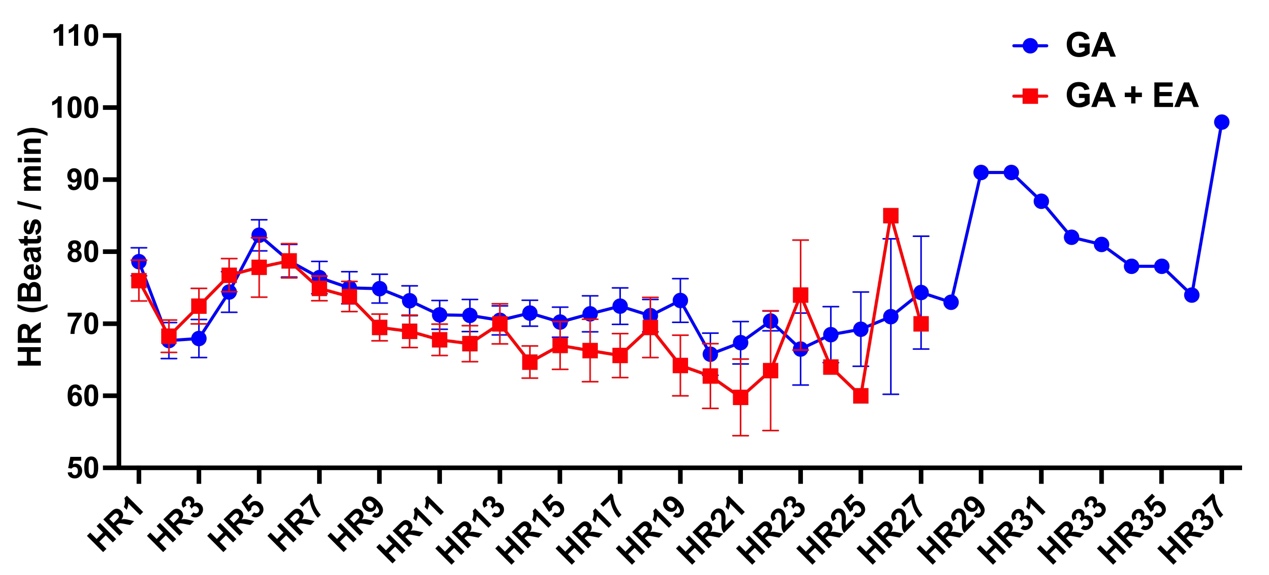


Supplement figure 2: Heat rate fluctuation between two groups

Data were recorded at 5min interval, MAP1 was the baseline value, MAP2 was the value right after induction. Most of the patient were finished their surgery within 200 mins. Four patients in GA group and 2 patients in the GA+EA group have exceeded 200 mins.
